# Synthesis and Characterization of Alginate Gel Beads with Embedded Zeolite Structures as Carriers of Hydrophobic Curcumin

**DOI:** 10.3390/gels9090714

**Published:** 2023-09-03

**Authors:** Gianluca Ciarleglio, Federica Cinti, Elisa Toto, Maria Gabriella Santonicola

**Affiliations:** Department of Chemical Engineering Materials Environment, Sapienza University of Rome, Via del Castro Laurenziano 7, 00161 Rome, Italy; gianluca.ciarleglio@uniroma1.it (G.C.); cinti.1751541@studenti.uniroma1.it (F.C.); elisa.toto@uniroma1.it (E.T.)

**Keywords:** alginate beads, curcumin, chitosan coating, drug delivery

## Abstract

Alginate-based beads containing a porous zeolite filler were developed as carriers of bioactive compounds with a hydrophobic nature, such as curcumin (Cur). Curcumin, a natural pigment extracted from the turmeric (Curcuma longa) plant, exhibits antioxidant, anti-inflammatory, anticarcinogenic, and antiviral properties. To enhance the bioavailability of the drug, curcumin needs to be encapsulated in a suitable carrier that improves its dispersibility and solubility. Commercial A-type zeolites (Z5A) were used as curcumin-binding agents and they were immobilized within the alginate gel beads by cross-linking in calcium chloride solution during an extrusion dripping process. The process parameters (alginate and CaCl_2_ concentrations, needle gauge, collecting distance) were optimized to fabricate beads with good sphericity factor and 1.5–1.7 mm diameter in their hydrated state. The chemical structure of the gel beads was assessed using FTIR spectroscopy, while their thermal stability was evaluated through differential scanning calorimetry (DSC) and thermogravimetric analysis (TGA). Due to the alginate matrix, the composite Alg/ZA5-Cur beads possess pH-responsive properties. In addition, the gel beads were modified by chitosan (CS) to enhance the stability and control the degradation behavior of the gel matrix. The swelling behavior and the degradation of the beads were analyzed in physiological solutions with different pH values. Results demonstrate the stabilizing and protective effect of the chitosan coating, as well as the reinforcing effect of the zeolite filler. This makes the pH-responsive alginate gel beads good candidates for the delivery of lipophilic drugs to specific inflammatory sites.

## 1. Introduction

Drug delivery systems play a crucial role in ensuring efficient and targeted delivery of therapeutic agents [1,2]. In recent years, researchers have been exploring innovative approaches to enhance drug delivery systems and improve therapeutic outcomes. In this perspective, biocompatible gels have attracted the attention of researchers for their applications as scaffolds, carriers, and controlled drug delivery systems able to respond to physiological stimuli, such as pH and temperature [3,4,5,6]. Gels are three-dimensional (3D) networks of cross-linked polymers capable of absorbing and retaining large amounts of water [7,8]. They are extensively researched in the biomedical field due to their unique properties, which include high permeability, controlled biodegradation, and the aptitude to provide support for engineered tissue structures [9]. In addition, due to their chemical versatility, hydrogels may be endowed with several functionalities, such as antimicrobial and anti-inflammatory properties, which are useful in the healing of infected wounds [10,11].

Alginate-based gels in the form of beads, microspheres, and nanoparticles are widely utilized in the biomedical field because they provide precise control over drug release rates and enable specific drug delivery to targeted therapeutic sites [12]. Additionally, these systems can expand or contract in response to changes in pH, allowing them to regulate the drug dosage at an optimal baseline level [13]. This prevents abrupt drug release and the development of multidrug resistance [14].

Alginate is a natural anionic linear polysaccharide composed of alternating blocks of α-L-guluronic and β-D-mannuronic acid residues linked by 1–4 bonds. Through intermolecular cross-linking with divalent cations such as Ca^2+^ ions, alginate can form a water-resistant gel network via ionotropic gelation at room temperature, allowing immobilization under mild and safe conditions. Alginate beads can encapsulate bioactive compounds, including phenolic compounds [15], lipophilic bioactive compounds [16], and proteins [17]. After drug release, the alginate degrades into water-soluble oligomers that are further metabolized and eliminated from the body due to their biodegradability.

pH-responsive alginate beads have great potential for delivering and controlling release of fat-soluble active principles, like curcumin. Curcumin is a polyphenolic compound isolated from the rhizome of the herb Curcuma longa. It exhibits low intrinsic toxicity and high therapeutic efficacy but suffers from challenges such as low water solubility, stability, and poor oral bioavailability, which limit its applications. Sreekanth Reddy et al. [18] fabricated sodium alginate/montmorillonite microbeads as a potential pharmacological vehicle for the extended release of curcumin. The microbeads were prepared using in situ ion exchange followed by ionotropic gelation. Wang et al. [13] synthesized sodium alginate/carboxymethyl chitosan-CuO hydrogel beads as a pH-sensitive carrier for the controlled release of curcumin. Through hemolysis, cytotoxicity and cell migration tests demonstrated that the composite beads have good biocompatibility and are a promising material for curcumin release in a gastrointestinal environment.

In some works, curcumin is loaded into inorganic materials, such as zeolites, to improve its stability. Zeolites are microporous inorganic crystalline materials with a three-dimensional framework comprising silicon, aluminum, and oxygen. They possess several advantages, including a well-defined pore structure, tunable base-acid sites, molecular sieve properties, and ion exchange capabilities. These materials are successfully used as carriers of various therapeutic compounds [19] and are characterized by high biocompatibility with various cell and tissue types [20]. Jiang et al. [21] synthesized curcumin-loaded zeolite Y nanoparticles embedded in electro-spun polycaprolactone and gelatin nano-fibers for the post-surgical treatment of glioblastoma.

This work introduces a promising approach for the transport of lipophilic drugs, which cannot be solubilized in aqueous media, using composite alginate/zeolite gel beads in which the active compound is immobilized within the zeolite porous structure. In fact, curcumin is a lipophilic molecule that is not directly soluble in the highly hydrophilic alginate beads. For this reason, in this work, curcumin was first loaded in porous zeolites before integration with the alginate-based gel carriers. An l-type zeolite molecular sieve (zeolite A) was employed as a drug binder for curcumin and dispersed in a sodium alginate solution to prepare pH-sensitive composite beads for controlling the release of curcumin. The alginate beads were fabricated using ionic gelation with extrusion-dripping method and the process parameters were optimized. The beads coated with chitosan were also prepared, to improve the response to pH. As reported in the literature [22,23,24], the application of a chitosan coating is helpful in improving the physicochemical stability of several types of drug carriers and can be used to control drug release, thus tailoring its bioavailability and efficacy. In this work, the beads’ modification by chitosan can be useful to prevent premature degradation of the beads in an acidic environment, such as that of the stomach, favoring the release of curcumin in the gastro–intestinal tract.

The alginate-based gel beads are extensively characterized using various techniques, such as optical microscopy, Fourier-transform infrared spectroscopy (FTIR), and differential scanning calorimetry (DSC). These characterization techniques provide insights into the structural morphology, chemical composition, and pH-responsive behavior of the gel beads, ensuring their suitability for drug delivery applications.

The synthesized gel beads offer pH-responsive release properties, enhanced stability, and improved therapeutic efficacy. This research holds significant potential for the development of advanced drug delivery systems with applications in various biomedical fields.

## 2. Results and Discussion

### 2.1. Optimization of Process Parameters

**Figure 1:**
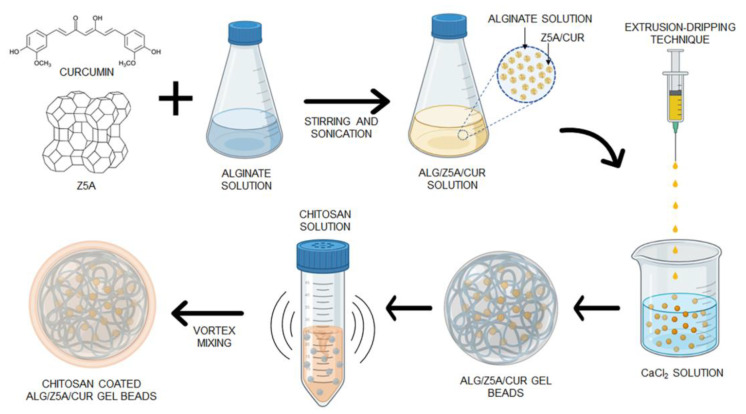
Schematic representation of the fabrication process of pH-responsive composite beads coated with chitosan by vortex mixing.

The fabrication process for Alg/Z5A/Cur beads was developed and optimized using the extrusion-dripping technique through ionic gelation. This process is schematically represented in Figure 1.

In preliminary tests, alginate beads were prepared by varying the concentration of alginate (1, 2, 4 wt%), the concentration of the CaCl_2_ solution (5, 10, 20 wt%), the collecting distance (1.6, 7, 10, 11.5 cm) and the needle gauge (23, 27, 30 G). Results are summarized in Table 1 and show that the smallest syringe needle (30 G) produced the smallest beads. However, the 4 wt% concentration of alginate was too viscous and difficult to extrude through a 30 G needle, while the 1 wt% concentration of alginate was not viscous enough, resulting in irregular bead shapes. Moreover, the size of the beads decreased as the concentration of the CaCl_2_ solution increased. This can be attributed to the shrinkage of the gel network as the concentration of Ca^2+^ ions increased [25], leading to smaller beads. However, the beads produced using the 20 wt% CaCl_2_ solution show an irregular, non-spherical morphology due to the high surface tension of the gelling bath during impact.

The best results, in terms of size and shape, were achieved using a 2 wt% alginate solution and a 10 wt% CaCl_2_ cross-linking solution. Each parameter was individually varied to assess its effect on the samples.

**Figure 2:**
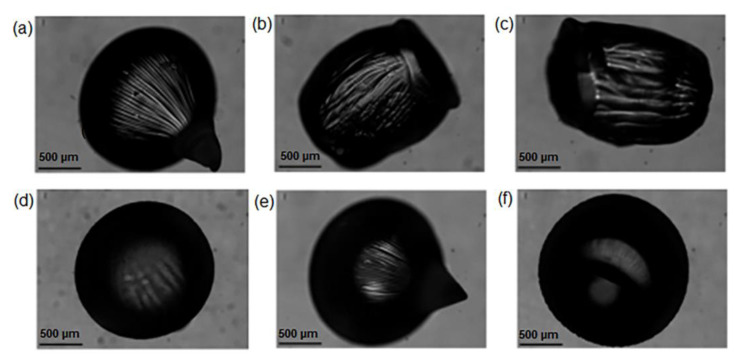
Beads shape at alginate concentrations of (**a**–**c**) 1 wt% and (**d**–**f**) 2 wt% using a collecting distance of (**a**,**d**) 7 cm, (**b**,**e**) 10 cm and (**c**,**f**) 11.5 cm.

The collecting distance is an important parameter that strongly depends on the viscosity of the alginate solution. Low-viscosity alginate solution droplets tend to flatten upon impact with the crosslinking bath, even at short collecting distances, which can lead to non-spherical beads. In this study, four different collecting distances were tested: 1.6 cm, 7 cm, 10 cm, and 11.5 cm. Figure 2 shows the effect of the collecting distance on the beads’ morphology at different alginate concentrations (1 wt% and 2 wt%) with a 10 wt% concentration.

The stirring speed during the polymerization process was investigated, and it was found that a stirring speed of 200 rpm resulted in elongated beads. As a result, the stirring speed was set to 0 rpm. The best results, in terms of size and shape, were achieved using a 2 wt% alginate solution, 10 wt% CaCl_2_ cross-linking solution, and a collecting distance of 7 cm.

**Figure 3:**
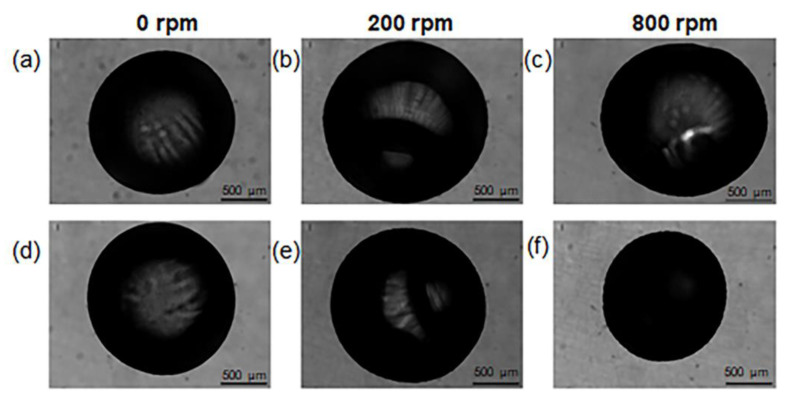
Evaluation of stirring speed on samples shape: (**a**–**c**) before chitosan coating and (**d**–**f**) after chitosan coating.

The chitosan coating was applied to the beads by transferring them into a chitosan solution and vortex-mixing the suspension at 800 rpm. During the coating process, the functional groups NH_3_^+^ from chitosan and COO^−^ from alginate form an ionic bond [16]. The effect of the stirring rate was further investigated. Stirring rates from 0 to 800 rpm were experimented with, and the resulting size of alginate beads was measured from the digital images (Figure 3).

The weight and diameter of beads before and after coating were compared, for each of the tested speeds. The results are listed in Table 2. As the mixing speed increases, the diameter of the beads decreases and the weight increases, which suggests a more compact structure for the beads obtained with a higher stirring speed (800 rpm).

The value of 800 rpm was selected for the preparation of small-size beads. Indeed, few works in the literature report that the coating applied through stirring may induce the shrinkage of beads [16,22]. In our case, this might be due to the diffusion of chitosan molecules inside the alginate matrix, which causes a partial collapse of the polymer network as a consequence of electrostatic neutralization. The chitosan diffusion is enhanced at higher mixing rates, resulting in a size decrease of the chitosan-modified beads.

For the fabrication of the composite beads, the alginate solution containing Z5A/Cur was dripped into the cross-linking solution. The process parameters used were those optimized in the preliminary experiment.

### 2.2. Morphological and Swelling Properties

**Figure 4:**
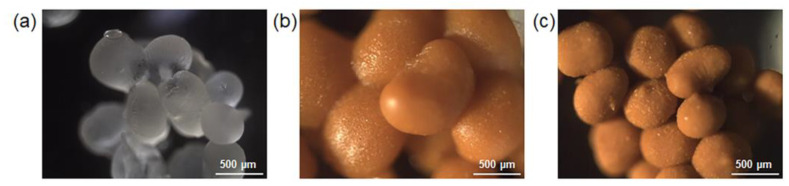
Optical microscopy images of dried (**a**) Alg, (**b**) Alg/Z5A/Cur, and (**c**) Alg/Z5A/Cur/CS beads.

The dry beads shown in Figure 4 were obtained by placing the gel beads in an oven at 50 °C for 18 h. They exhibit a spherical shape, with a compact structure. The orange-yellow color of curcumin pigment was observed in Alg/Z5A/Cur with and without chitosan coating (Figure 3a,b). When comparing the composite beads, it can be observed that those with a chitosan coating appear to be more compact and regular in structure compared to those without the coating.

Dimensional analysis and sphericity factor (SF) evaluation were conducted on swollen and dried beads using optical microscopy. The mean diameter values and SF of the beads are reported in Table 3. The analysis revealed that the composite beads are slightly larger in size compared to the unloaded ones. The use of gel beads with dimensions of the order of 1–2 mm in their swollen state has several advantages. First, the fabrication process by extrusion dripping at this length scale is easier to handle and allows for the production of a large number of beads in a relatively short time (few minutes). This reduces costs and improves the overall process throughput. In addition, beads with larger sizes are easier to handle and the tendency to form aggregates is minimal, thus eliminating the need for specialized equipment.

For all types of hydrated beads, the modification by chitosan (at a vortex stirring rate of 800 rpm) results in a statistically significant decrease in diameter when compared to the pure alginate beads (*p* < 0.05). Additionally, the sizes of the beads decrease significantly upon drying, which can be attributed to their high-water content, as shown in Figure 3. For the dried beads, statistically significant differences in mean diameter were observed between the unloaded Alg/CS and the curcumin-loaded Alg/Z5A/Cur/CS beads (*p* = 0.0177). All types of beads have an SF ≤ 0.05, indicating their close approximation to a spherical shape, except for the dry alginate spheres with and without coating. The use of spherical beads might have several advantages. The spherical shape of the beads can provide a more uniform release of the drug, which occurs through surface erosion of the gel beads. This might facilitate the control of the drug release over a defined period, thus improving therapeutic results. In addition, this geometry maximizes the ratio of volume to surface area resulting in increased loading capacity. Finally, the symmetrical nature of the spherical beads increases their structural strength, reducing the risk of deformation or breakage along one direction during handling.

**Figure 5:**
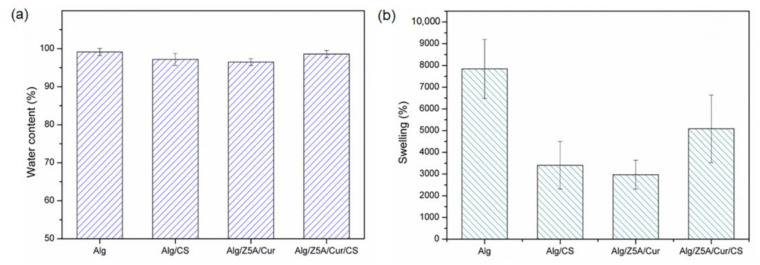
(**a**) Water content and (**b**) swelling ratio of different types of beads in ultrapure water at T = 25 °C.

Figure 5 shows the water content (%) (Figure 5a) and the swelling ratio (%) (Figure 5b) of gel beads at room temperature. The water content is not significantly different in the four types of samples, and all beads show a water content of over 96%. The high-water content causes the shrinkage of the polymer mesh when dried, as shown in Table 3.

### 2.3. Thermal Analysis

**Figure 6:**
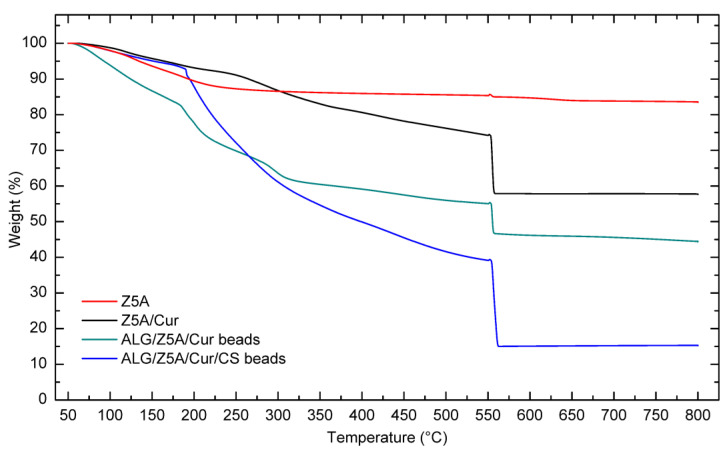
TGA curves of Z5A, Z5A/Cur, and Alg/Z5A/Cur beads with and without chitosan coating.

The thermal stability of the compounds used and of the gel beads was investigated by thermogravimetric analysis (TGA). Figure 6 shows the TGA curves of Z5A, Z5A/Cur, and Alg/Z5A/Cur beads with and without chitosan coating. 

The TGA curve of Z5A indicates a mass loss that can be attributed to the release of water trapped in the nano-porous zeolites. Z5A samples loaded with Cur exhibit similar behavior to that of pure zeolites. Additionally, above 250 °C there is a mass loss associated with the thermal degradation of curcumin. The thermogram of the Alg/Z5A/Cur beads shows three distinct thermogravimetric effects. In the low-temperature range (50–180 °C), an initial mass loss occurs due to the desorption of water trapped in the zeolite cavities and water bound to the alginate. In the intermediate temperature range (180–300 °C), a mass loss is observed, which can be attributed to the breakdown of the alginate backbone and the thermal degradation of curcumin. In the higher temperature range (300–550 °C), mass loss occurs due to the thermal degradation of both alginate and curcumin. 

The curve of the Alg/Z5A/Cur/CS beads exhibits a similar trend to those without chitosan coating. However, there is a higher mass loss compared to the previous case, as indicated in Table 4.

**Figure 7:**
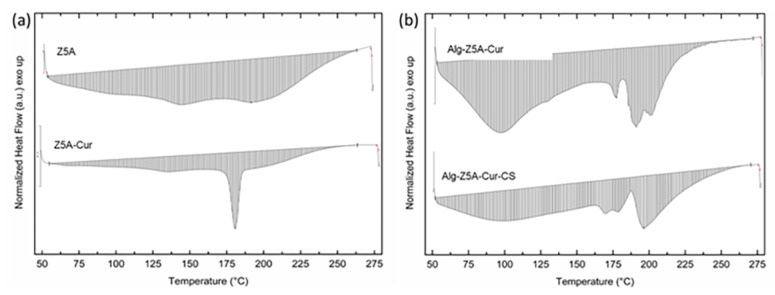
DSC thermograms of (**a**) Z5A and Z5A/Cur, (**b**) Alg/Z5A/Cur beads, and Alg/Z5A/Cur/CS beads.

Differential scanning calorimetry (DSC) on pure zeolites, Z5A/Cur, and Alg/Z5A/Cur beads, with and without chitosan coating was performed in the temperature range 50–270 °C. The thermograms are reported in Figure 7. Empty Z5A exhibited no significant thermal events within the temperature range examined. The DSC thermogram of Z5A/Cur exhibited an endothermic peak at around 181 °C, attributed to the melting of curcumin (curcumin melting point 183 °C), which confirms previous literature data [26]. The peak observed is slightly shifted compared to the peak referring to pure curcumin, which may indicate the crystallization of the drug in the nanopores of the zeolite [19,27]. The curve of the Alg/Z5A/Cur beads shows a broad endothermic peak at 95 °C, probably due to water bonded to the structure via hydrogen bonding, and the endothermic peak around 177 °C was associated with curcumin melting. The asymmetric shape of the latter peak may suggest the fusion and decomposition of the bead network. The curve for Alg/Z5A/Cur/CS beads exhibited an endothermic event with an asymmetric peak between 170 and 180 °C, and the peak observed at 196 °C could be attributed to the interaction between alginate and chitosan.

### 2.4. FTIR Analysis

**Figure 8:**
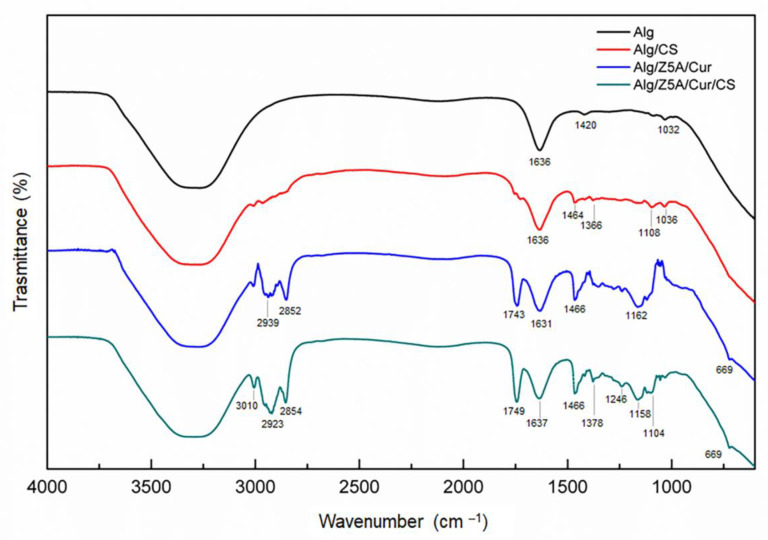
FTIR spectra of different types of beads. Data are offset for clarity.

Figure 8 shows the spectra of different types of swollen gel beads. All spectra exhibit a broad absorption band at approximately 3400–3200 cm^−1^ and a band at 1636 cm^−1^ corresponding to the stretching of O–H and asymmetric stretching vibration of alginate COO groups, respectively. The antisymmetric stretching of the alginate carboxyl group (1420 cm^−1^) and the stretching vibrations of C– OH side groups (1032 cm^−1^) can be observed in alginate beads. In alginate beads with chitosan coating, the peaks at 1464 cm^−1^ and 1366 cm^−1^ are caused by the deformation of the C– H and CH_2_OH groups of chitosan [28]. The peak at 1108 cm^−1^ falls within the band of aliphatic ethers and could be attributed to the antisymmetric stretching of the C–O–C bridge [29]. The presence of new peaks, and the displacement of others, confirm the interaction between the alginate and chitosan. 

The spectra of composite beads show peaks at 2939 cm^−1^ and 2852 cm^−1^, which are due to C–H stretching [30] and the deformation of the curcumin methyl group [27], respectively The peak at 1743 cm^−1^ is attributed to the stretching of the C=O group of curcumin [31,32]. The absorption at 1466 cm^−1^ and 1162 cm^−1^ is attributed to the vibration of the C–H group and the deformation of the aromatic ring. In the fingerprint zone, the stretching vibration of the Si–O–Al bridge at 669 cm^−1^, typical of zeolites [33], confirms that the Z5A was correctly dispersed into the alginate beads. In the spectra of chitosan-coated composite beads, the peak at 1378 cm^−1^ is due to the symmetrical deformation of methyl groups of chitosan, and the peaks at 1246 cm^−1^ and 1158 cm^−1^ are attributable to the skeletal deformation (C–CHO), probably superimposed on the C–O stretching of the aromatic ring of curcumin [34]. The absorption at 1104 cm^−1^ is caused by the overlapping of the C–O and C–O–C stretching of alginate and chitosan [35].

### 2.5. Swelling Kinetics and Degradation Tests

Swelling kinetics and degradation tests were conducted to evaluate the pH response of the beads and the stabilizing effect of chitosan coating. 

**Figure 9:**
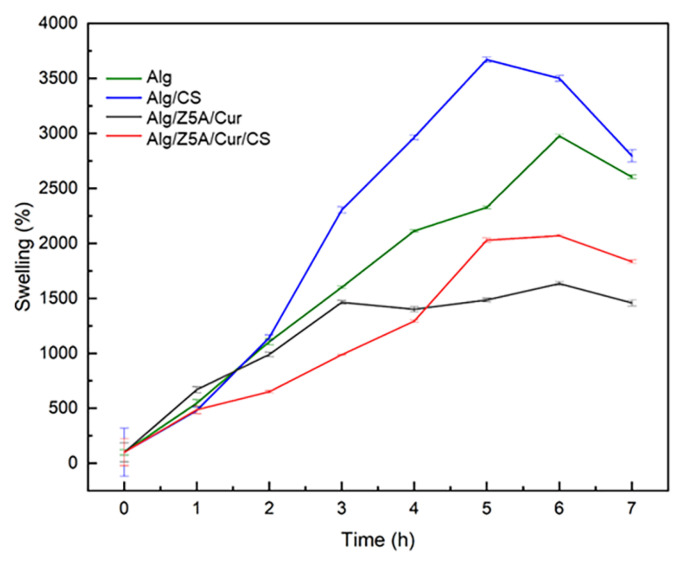
Swelling kinetics of different types of beads in PBS solution (pH = 7.4). Test performed at a temperature of 25 °C.

A preliminary test was conducted on empty, filled beads with and without chitosan coating immersed in a PBS solution with a pH of 7.4 at room temperature (25 °C) for 6 h (Figure 9). All types of beads show a high swelling ratio, beads with chitosan coating exhibiting a higher swelling ratio after 5 h than those without, which is due to the hydrophilic nature of chitosan [36].

In addition, the empty beads exhibit a higher swelling ratio compared to the loaded ones; this could be attributed to the zeolites encapsulated within the three-dimensional structure of the gel, which limit the extension of the polymer chains [37,38]. 

**Figure 10:**
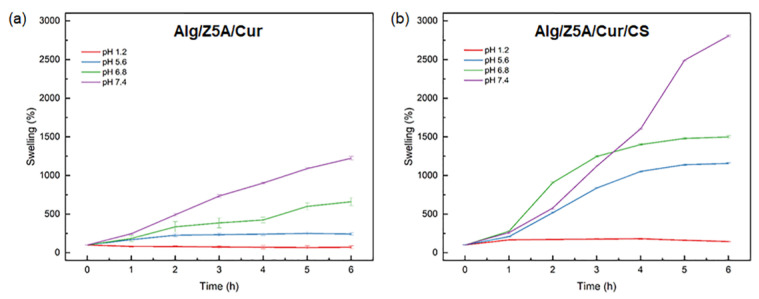
Swelling kinetics at different pH values of Alg/Z5A/Cur beads (**a**) with and (**b**) without chitosan coating. Test performed at a temperature of 37 °C.

To assess the effect of the pH value on the swelling ratio, swelling kinetics was performed on Alg/Z5A/Cur beads with and without chitosan coating in simulated gastric fluid (SGF, hydrochloric acid pH = 1.2), acid tumoral environment (pH = 5.6), simulated intestinal fluid (SIF, phosphate buffer pH = 6.8) and simulated colon fluid (SCF, phosphate buffer pH = 7.4) for 6 h at 37 °C. Figure 10 shows the trends for the swelling kinetics at different pH values of Alg/Z5A/Cur beads (a) with and (b) without chitosan coating. It can be observed that the swelling ratio is dependent on the pH value. In particular, for both types of beads, as the pH value increases, the swelling ratio increases, with a minimum at pH 1.2 (SGF) and a maximum at pH 7.4 (SCF), due to the formation of a less rigid network.

**Figure 11:**
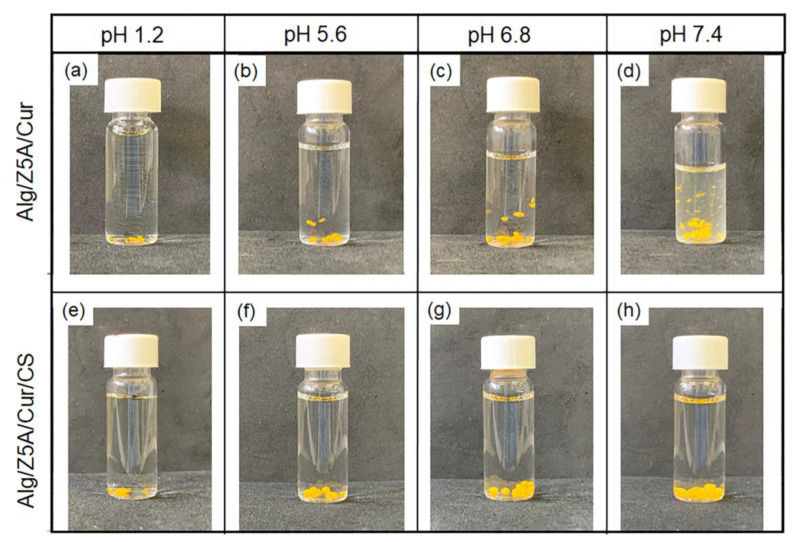
Degradation tests performed at 37 °C in PBS at pH = 1.2 (**a**,**e**), pH = 5.6 (**b,f**), pH = 6.8 (**c**,**g**), and pH = 7.4 (**d**,**h**) on Alg/Z5A/Cur beads without coating (**a**–**d**) and on Alg/Z5A/Cu beads with chitosan coating (**e**–**h**). Degradation evaluated after 6 h.

The swelling mechanism is pH-dependent, so it is linked to the deprotonation of the carboxyl groups of the alginate. At pH = 1.2, the deprotonation percentage of the carboxyl group, calculated using the Henderson–Hasselbalch equation [39], is 0.004%. Consequently, there is poor electrostatic repulsion between the alginate chains, resulting in shrinkage of the polymer network [40]. This result is confirmed in degradation tests showing unaltered beads at pH = 1.2 (Figure 11a,e). By increasing the pH to 5.6 (pH > pK_a_ (alg)), the concentration of -COO^−^ increases, increasing electrostatic repulsion between the carboxylate anions and the swelling ratio [41]. In the Alg/Z5A/Cur/CS beads, the amino groups of the chitosan are in their protonated form (NH_3_^+^) as the pH < pKa (CS), so degradation does not occur (Figure 11f).

**Figure 12:**
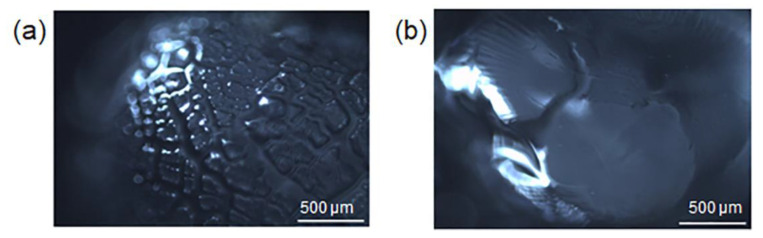
Optical microscopy images of swollen beads after 48 h in PBS solution (pH = 7.4): (**a**) Alg and (**b**) Alg/CS beads.

At pH = 6.8, the deprotonated carboxyl groups increase, and the protonated amines decrease. In Alg/Z5A/Cur/CS beads, the interaction between chitosan and alginate decreases and the swelling increases due to the electrostatic repulsion of -COO^−^. At pH =7.4, the -COO^−^ increases and the amino groups are almost all deprotonated (NH_2_): the interaction between the two polysaccharides is weak and the swelling increases due to the increase of the electrostatic repulsion. Degradation at pH = 7.4 is shown in Figure 11d, whereas it is delayed in beads with the chitosan coating (Figure 11h). This result is due to the protective effect of the chitosan coating, as can be seen in Figure 12, which shows the surface of the beads with and without coating after 48 h in PBS solution (pH = 7.4). Extensive surface erosion of Alg beads can be appreciated (Figure 12a), while the Alg/CS beads show minor cracking phenomena (Figure 12b).

## 3. Conclusions

Composite alginate/zeolite gel beads with pH-responsive properties were developed as carriers for lipophilic drugs, such as curcumin. The drop extrusion technique was used to crosslink the beads’ polymer network and the process parameters were optimized. The properties and the swelling behavior of the composite beads were studied using various methods. FTIR spectroscopy verified Z5A/Cur incorporation in the alginate network. Optical microscopy showed round gel beads (SF < 0.05) with a compact structure, smaller when chitosan-coated due to electrostatic effects. Swelling ratio analysis indicated high water content (>96%), which is compatible with soft tissue microenvironments. Thermal analysis (TGA, DSC) confirmed curcumin loading in zeolites. The Alg/Z5A/Cur gel beads demonstrated different degrees of swelling and stability depending on the environment pH, with lower swelling in acidic conditions, such as in simulated gastric fluid (SGF). Beads remained stable in acidic environments and started degradation at pH 5.6. The chitosan modification further improved stability of the beads in an acidic environment. Results in terms of pH-responsive behavior and stability make Alg/Z5A/Cur/CS beads promising carriers for lipophilic drugs in specific inflammatory sites.

## 4. Materials and Methods

### 4.1. Materials

Alginic acid sodium salt, chitosan, calcium chloride (CaCl_2_) dihydrate, acetic acid, curcumin, and phosphate buffered saline were purchased from Sigma-Aldrich (Schnelldorf, Germany). Zeolite 5A, the calcium-exchanged form of zeolite Linde type A, was obtained in bead form from Zeochem AG (Rüti, Switzerland). Ethanol was purchased from VWR Chemicals (Radnor, Pennsylvania). Deionized water (resistivity 18.2 MΩ⋅cm) was produced by a Direct-Q3 UV water purification system (Millipore, Molsheim, France) and used in all preparations.

### 4.2. Fabrication of Alginate Gel Beads

Sodium alginate was added to ultrapure water at 1–4 wt% concentrations and mixed for 2 h using a magnetic stirrer (C-MAG HS7, IKA, Staufen, Germany). Beads were produced using the extrusion-dripping method, where the prepared alginate solutions (1, 2, and 4 wt%) were added dropwise using a 1 mL syringe into a CaCl_2_ aqueous solution. The crosslinking time was set at 30 min, followed by rinsing the beads three times in deionized water and placing them in a beaker containing ultrapure water. Preliminary tests were conducted to optimize the process parameters. In particular, the following parameters were varied: alginate concentration (1, 2, and 4 wt%), collecting distance (1.6 cm, 7 cm, 10 cm, and 11.5 cm), and needle gauge (27 G, 30 G). The chitosan-coated beads were prepared by vortex mixing in a 0.5 wt% chitosan solution for 30 min, then rinsed and stored in deionized water. Three different mixing speeds were evaluated during the coating process: 0 rpm, 300 rpm, and 800 rpm.

### 4.3. Preparation of Chitosan Coated Alg/Z5A/Cur Gel Beads

Curcumin was loaded in the porous zeolites following a previously published protocol [27]. Briefly, 0.25 g of 5A zeolites (Z5A) were ground into powder using a ceramic mortar. The resulting powder was then dehydrated by placing it in an oven at 120 °C overnight. This process ensures the removal of water inside the pores that could interfere with the adsorption process. Subsequently, the zeolites were incubated with a solution of curcumin in ethanol (0.05 mol/L) and stirred for 48 h. The ethanol/Z5A/Cur solution was then centrifuged for 10 min at 6000 rpm and dried in an oven at 60 °C for 12 h to remove any residual solvent. The loading efficiency (LE%) of the zeolites Z5A was determined following our previously published protocol [27]. For an incubation solution with curcumin concentration of 0.05 mol/L, the LE% was 51%.

An amount of 0.2 g of Z5A-Cur was added to 10 mL of alginate solution to prepare the composite beads. The Alg/Z5A/Cur solution was stirred for 15 min and then sonicated for 45 min to achieve a homogeneous solution.

Composite beads were fabricated using the extrusion-dripping method, where the Alg/Z5A/Cur solution was added dropwise into a CaCl_2_ aqueous solution, following the parameters optimized in the initial steps of the work. The beads were further optimized with a chitosan coating.

### 4.4. Surface Morphology and Dimensional Analysis

A Leica DMLP polarized microscope equipped with 5×, 10×, and 20× objective lenses was utilized to analyze the surface morphology and particle size of the beads. The average size of the beads was measured using ImageJ software (version 1.53). The sphericity factor (SF) was determined using the following equation [42]:

(1) $$SF=\frac{D_{1}-D_{2}}{D_{1}+D_{2}}$$

where *D_1_* represents the larger diameter of the beads, while *D_2_* is the smaller one. When *SF* ≤ 0.05, the shape of the matrix can be approximated to a sphere. Data were analyzed using a t-test to determine any statistical difference. The significance was determined at the 95% confidence level.

### 4.5. Thermal Characterization

The thermal properties and thermogravimetric analysis (TGA) of zeolite, curcumin-loaded zeolite, and composite bead samples were investigated using a TGA/DSC 1 STARe instrument (Mettler Toledo, Greifensee, Switzerland). For the thermal analysis, dry samples weighing approximately 10 mg were measured in the temperature range of 50 °C to 270 °C at a heating rate of 10 °C/min under a nitrogen flow of 40 mL/min. An empty aluminum pan served as the reference cell.

TGA was performed on dry samples weighing 5–10 mg to confirm the successful surface modification of the zeolites and to evaluate the thermal behavior of the beads. The analysis was conducted in the range 50–800 °C with a heating rate of 10 °C/min. A nitrogen atmosphere was used up to 550 °C, after which the nitrogen flow was replaced by oxygen to complete combustion of the organic compounds. Three replicates were run for each type of sample. The STARe software provided with the instrument was used for curve analysis.

### 4.6. FTIR Analysis

Fourier transform infrared (FTIR) spectra of beads were recorded using a Nicolet^TM^ Summit/Everest^TM^ Spectrometer (Thermo Fisher Scientific, Waltham, Massachusetts, US) equipped with a zinc selenide attenuated total reflectance (ATR) accessory. IR spectra were acquired in the region of 4000–500 cm^−1^, with a resolution of 4 cm^−1^, and each spectrum was acquired by averaging 32 scans. To account for the background interference from the surrounding air, the data were corrected by subtracting an air background spectrum.

### 4.7. Swelling Properties

To determine the swelling ratio and water content, the gel beads were first completely hydrated in deionized water and then dried at 50 °C for 18 h. The swelling ratio and water content were calculated using the following equations [43]:

(2) $$Swelling\;Ratio\left(\%\right)=\frac{W_{swollen}}{W_{dry}}\times 100$$

(3) $$\mathrm{Water}\;\mathrm{content}\;(\%)=\frac{W_{swollen}-W_{dry}}{W_{swollen}}\times 100$$

W_swollen_ is the weight of the gel in the equilibrium swelling state, and W_dry_ is the weight of the fully dried gel. The test was repeated on 5 samples for each bead type and the results averaged. 

### 4.8. Swelling Kinetics and Degradation Test

Swelling kinetics and degradation tests were conducted to evaluate the pH response of the beads and the stabilizing effect of chitosan coating. Tests were performed on 3 mg of dried beads (18 h at 50 °C) immersed in 4 mL of solution. A preliminary test of swelling kinetics was conducted on all types of gel beads in PBS solution (pH = 7.4) for 6 h. The swelling kinetics were studied for Alg/Z5A/Cur beads with and without chitosan coating in simulated gastric fluid (SGF, hydrochloric acid pH = 1.2), acid tumoral environment (pH = 5.6), simulated intestinal fluid (SIF, phosphate buffer pH = 6.8) and simulated colon fluid (SCF, phosphate buffer pH = 7.4) for 6 h at 37 °C. Degradation tests were performed at different pH values in PBS solution, monitoring the beads’ behavior after 6 h.

## Figures and Tables

**Table 1:** The mean diameter of alginate beads with different process parameters.

Alginate Solution (wt%)	CaCl_2_ Crosslinking Solution (wt%)	Needle Gauge (G)	Stirring Rate (rpm)	Collecting Distance (cm)	Mean Diameter (mm)
1	10	30	0	7	1.68 ± 0.04
	10	30	0	10	1.80 ± 0.31
	10	30	0	11.5	1.63 ± 0.05
2	5	27	0	1.6	2.35 ± 0.30
	10	27	0	1.6	1.95 ± 0.30
	10	30	0	1.6	1.56 ± 0.10
	10	30	0	7	1.54 ± 0.05
	10	30	0	10	1.68 ± 0.12
	10	30	0	11.5	1.66 ± 0.10
	10	30	200	1.6	1.59 ± 0.09
	10	30	200	7	1.67 ± 0.11
	10	30	200	10	1.68 ± 0.10
	10	30	200	11.5	1.66 ± 0.10
	20	30	0	7	1.59 ± 0.05
4	5	23	0	1.6	2.42 ± 0.20
	10	27	0	1.6	2.42 ± 0.30
	10	27	0	1.6	2.47 ± 0.07

**Table 2:** Variation in diameter (ΔD) and weight (ΔWt) of chitosan-coated alginate beads after vortex mixing at different stirring speeds.

	0 rpm	200 rpm	800 rpm
ΔD	0.04 ± 0.07	−0.02 ± 0.10	−0.10 ± 0.20
ΔWt	0.9 ± 0.1	1.1 ± 0.2	1.2 ± 0.2

**Table 3:** Mean values for diameter and sphericity factor (SF) of swollen and dry beads.

	Swollen		Dry	
Beads Type	D_s_ (mm)	SF_s_	D_d_ (mm)	SF_d_
Alg	1.54 ± 0.05	0.02 ± 0.03	0.50 ± 0.08	0.06 ± 0.04
Alg/CS	1.51 ± 0.02	0.03 ± 0.02	0.48 ± 0.08	0.06 ± 0.08
Alg/Z5A/Cur	1.71 ± 0.08	0.03 ± 0.02	0.82 ± 0.12	0.03 ± 0.05
Alg/Z5A/Cur/CS	1.67 ± 0.05	0.03 ± 0.02	0.52 ± 0.03	0.03 ± 0.03

**Table 4:** Weight loss and residue of Z5A, Z5A/Cur, and Alg/Z5A/Cur beads with and without chitosan coating. Standard deviation of data (three replicates) is below 2%.

Sample Type	Weight Loss at 800 °C (%)	Residue at 800 °C (%)
ZA5	15.80	82.41
ZA5/Cur	41.52	56.86
Alg/Z5A/Cur beads	41.89	37.49
Alg/Z5A/Cur/CS beads	83.19	15.10

## Data Availability

Data are available from the corresponding author upon reasonable request.
